# Sandwich-structured PVDF/protein piezoelectric hydrogel regulates osteogenesis and adipogenesis through glycerophospholipid metabolism in osteoporotic inflammatory bone defects

**DOI:** 10.1016/j.mtbio.2026.103770

**Published:** 2026-10-21

**Authors:** Weisin Chen, Shuyan Zhang, Lei Huang, Yukai Huang, Minhao Liang, Xin Liu, Ning Wang, Juan Li, Haifeng Liang, Dihan Su, Zenggan Chen, Dong Zhou, Yulin Li, Jian Zhang, Yan Su, Yutong Gu, Libo Jiang

**Affiliations:** a Department of Orthopaedics, Zhongshan Hospital, Fudan University, Shanghai, China; b Department of Orthopedic Surgery, Shanghai Sixth People's Hospital, Affiliated with Shanghai Jiao Tong University School of Medicine, Shanghai, 200233, China; c The State Key Laboratory of Bioreactor Engineering and Key Laboratory for Ultrafine Materials of the Ministry of Education, East China University of Science and Technology, Shanghai, 200237, China; d Orthopaedic Residency Program, Huashan Hospital, Fudan University, Shanghai, China; e Department of Orthopaedics, Shanghai Geriatric Medical Center, Fudan University, Shanghai, China; f Division of Spine, Department of Orthopaedics, Tongji Hospital, Tongji University School of Medicine, Shanghai, 200065, China; g Department of Geriatrics and Gerontology, Huadong Hospital, Fudan University, Shanghai, 200040, China; h Osteoporosis Specialized Prevention and Treatment Center, Xiamen, 361012, China

**Keywords:** Piezoelectric, PVDF, Silk fibroin, Osteogenesis, Adipogenesis, Multi-omics

## Abstract

Osteoporosis can occur when age-related ovarian dysfunction leads to estrogen deficiency. When bone mineral content decreases, fragile bones are susceptible to bone defects. This estrogen deficiency causes differentiation of mesenchymal cells to shift towards adipogenesis and reduced osteogenesis, which culminates in nonunion. With the potential to modulate cell differentiation, electrical stimulation is promising for osteoporosis defects complicated by an inflammatory environment. A sandwich-structured silk fibroin hydrogel (RSF) with three layers of polyvinylidene fluoride (PVDF) membrane with many β-crystalline phases and MXene for electrical conductance was fabricated, generating voltages up to 500 mV to leverage the implanted bone grafts for a sustainable source of electrical stimulation. Combined transcriptomic and metabolomic analyses revealed that piezoelectric stimulation promoted osteogenesis and suppressed glycerophospholipid metabolism by upregulating lipid degradation–related genes. Specifically, Pla2g4a was critical to the hydrolysis of phospholipids, as shown by Sudan black B staining in rBMSCs. Finally, we created femoral defects in osteoporotic rats and implanted MXene/PVDF/RSF hydrogel to promote osteogenesis and reduce adipogenesis, as shown by micro-CT and immunostaining. As a result, piezoelectric MXene/PVDF/RSF hydrogel effectively redirected bone-fat balance and overcame the inflammatory environment in osteoporotic bone defects, serving future clinical applications.

## Graphical abstract

**Figure:**
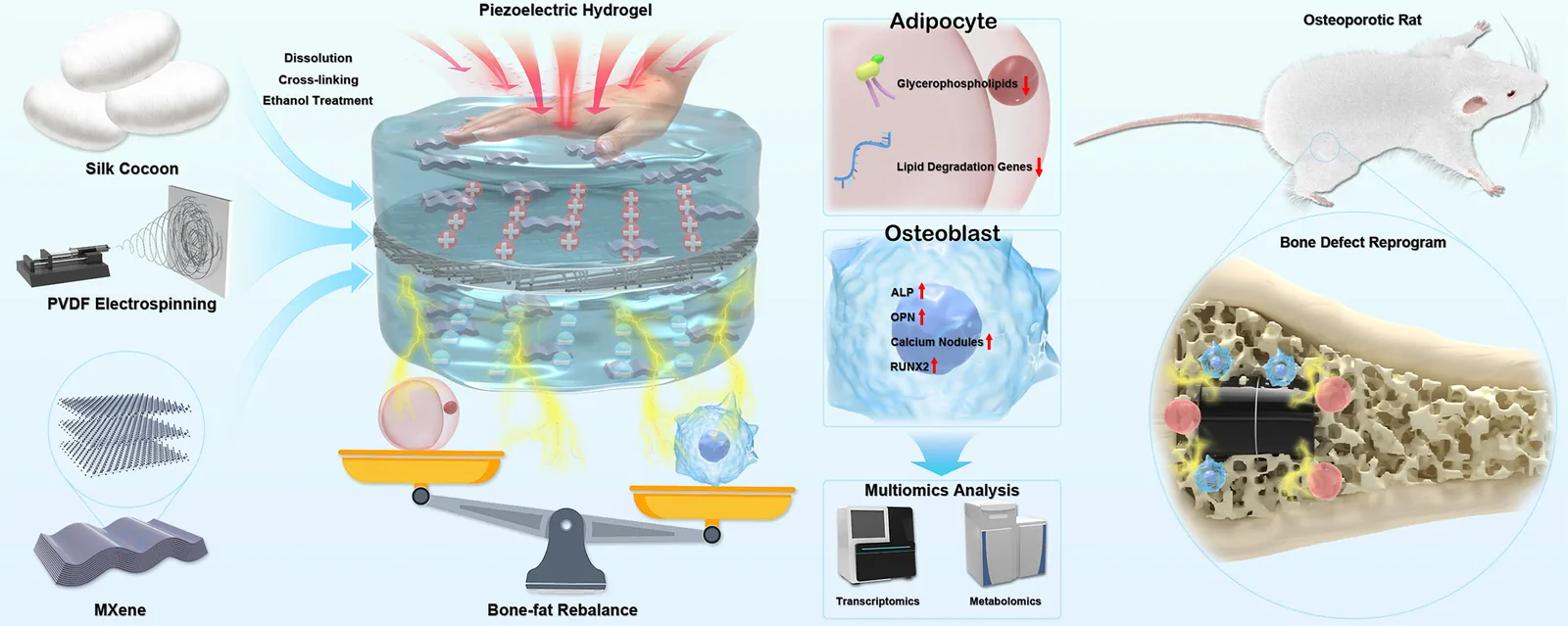

## 1 Introduction

Osteoporosis is characterized by loss of mineral content of bone due to an imbalanced bone-fat ratio facilitated by estrogen deficiency and is estimated to affect 18-20% globally [1], [2]. When women age, the estrogen secreted by the granulosa cells that line the ovarian follicles declines gradually, leading to menopause and overly activated osteoclasts. The resorbed bone was replaced by adipose tissue, resulting in lower mineral content, mainly hydroxyapatite, and decreased mechanical strength. Fracture can be inflicted by minor force and evolve to bone defects as the shifting osteogenesis-adipogenesis promotes adiposity [3], which cannot effectively heal the fracture gap and remodel it into mature bone. The healing process is also complicated by inflammation and cytokines, such as TNF-α, delaying the bone healing and contributing to nonunion [4]. Recently, electrical cues have been found to modulate mesenchymal cell differentiation, promote cell growth, and enhance cellular maturation and marker expression [5], [6]. However, the effects of electrical stimulation on the tilted bone-fat balance in an inflammatory environment have not been studied.

Electrophysical activities are integral to the normal functioning of numerous organs in mammals, and electrical stimulation is widely employed as a conservative treatment or as a rehabilitation measure to accelerate post-surgical healing [7], [8]. The electrical properties of bones and the mechanisms underlying fracture healing have been extensively documented in several studies [9], [10]. When stress is applied to bone, electrical currents are generated, concurrently inducing ion flux in the regenerating bone cells and altering downstream signaling molecules [11]. Electrical stimulation has been observed to influence the fate of various cell types, including the induction of osteogenesis from adipose-derived stem cells [12], [13]. Therefore, electrical stimulation coupled to stress via the piezoelectric effect represents a promising therapeutic approach [14]. Recently, several studies have developed piezoelectric implants by using amino crystals, biodegradable polymers, and piezoelectric ceramics, which can generate electrical stimulation in response to knee movements or external ultrasound [15], [16], [17]. For instance, Chorsi et al. combined glycine with poly(caprolactone) (PCL) films to enhance elasticity and ductility. However, PCL is not electrically conductive, and only the glycine grains exposed on the surface are effective. Moreover, these amino crystals dissolve rapidly upon contact with synovial fluids [15]. Poly(l-lactic acid) (PLLA) membranes with coaxially aligned fibers are another biodegradable material that generates a potential difference and stimulates osteogenesis, although the underlying biological mechanism remains poorly understood [17], [18]. Barium titanate nanoparticles combined with hyaluronic acid could respond to ultrasonic waves and facilitate stem cell adherence [16]. However, these nanoparticles tend to dissipate easily when embedded in osteochondral defects, especially under force. Additionally, they may grind the surfaces of the knee cartilage and induce osteolysis [19], [20]. Therefore, there is an urgent need to develop new materials capable of coupling mechanical stress with piezoelectric stimulation to promote bone tissue regeneration.

Polyvinylidene fluoride (PVDF) is a dielectric polymer that generates an electrical potential through mechanical stress. The high dipole moment of PVDF (μv = 7.0 × 10^−30^ cm) is attributed to electronegative fluoride and hydrogen atoms, making it a promising candidate for energy harvesting [21], [22]. PVDF exhibits multiple crystalline phases, among which the β-phase is considered the most piezoelectric. Several processing methods, such as electrospinning, increase the β-phase content [23], [24]. A study demonstrated minimal foreign body reaction after six-month implantation, suggesting that PVDF is suitable for in vivo use, particularly for long-term bone regeneration [25]. Wang et al. implanted a PVDF-based piezoelectric generator subcutaneously and detected an electrical potential of 0.26 V through muscle movement [26]. Moreover, the mechanical and electrical properties of PVDF can be tailored to specific needs through proper processing and combination, enabling biomedical applications such as sensor patches [27], [28]. Despite their excellent piezoelectric properties, PVDF membranes have certain limitations. The hydrophobic nature of PVDF hinders cell adhesion, which is crucial for bone regeneration. Improving hydrophobicity through copolymerization may compromise the piezoelectric properties [29], [30]. Additionally, the relatively long degradation time and the soft, flat nature of PVDF membranes make them unsuitable for tissue ingrowth and load support as bone fillers. Combining PVDF membranes with electrically conductive soft materials allows more efficient stress transfer from bone to the PVDF membrane, and the electric charges can be evenly distributed on the surface of the soft material in close contact with biologically active growing tissues, thereby enhancing regeneration.

Silk fibroin (RSF) is renowned for its biocompatibility and versatility, although it is generally considered bioinert. Our previous and recent work from Mao et al. has shown that silk fibroin-based hydrogels are excellent materials for skull defects, as they can be molded into diversified shapes and withstand thousands of compression cycles [31], [32]. Moreover, the electrical conductivity of RSF can be achieved by combining it with MXene for in vivo electronics [33]. In this study, MXene as a conductive substance was combined with PVDF as a source of piezoelectricity to facilitate electrical stimulation (Fig. 1). In vitro, the piezoelectricity successfully induced osteogenesis and reduced adipogenesis by enhancing glycerophospholipid degradation. In vivo, the inflammatory defect in the osteoporotic rat femur was implanted with piezoelectric MXene/PVDF/RSF hydrogel, and the adipose tissue was decreased, in contrast to increased bone, favoring bone healing.

**Fig. 1:**
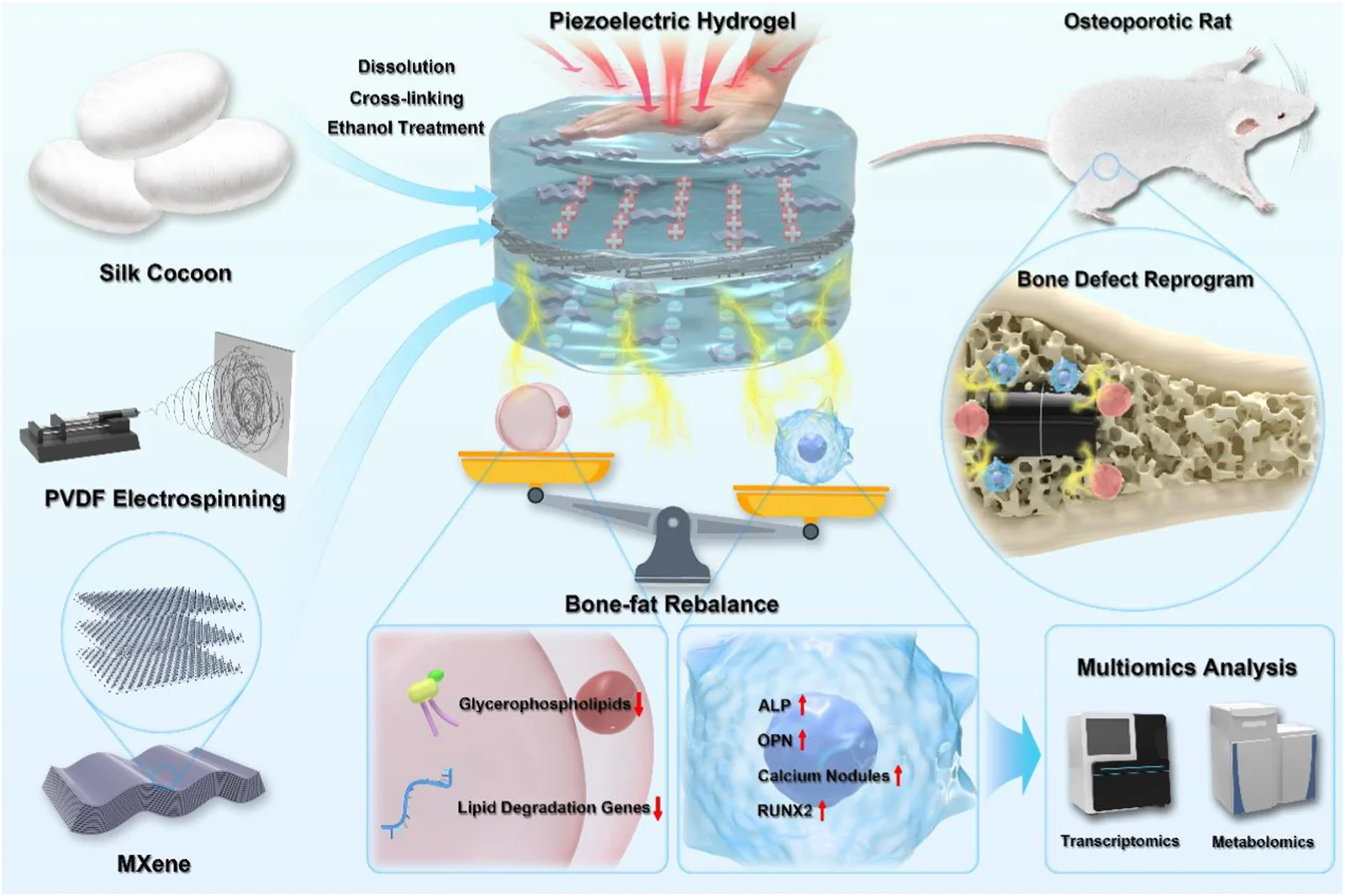
A high-power piezoelectric hydrogel was fabricated by integrating an electrospun PVDF membrane with an RSF hydrogel containing MXene. The piezoelectric properties of the hydrogel were characterized, generating voltages up to 500 mV under mechanical compression. The effects of piezoelectric stimulation on osteogenesis and adipogenesis in inflammatory bone cells were evaluated using a custom-made compression device. High-throughput multi-omics analysis revealed that glycerophospholipid metabolism was the primary pathway altered, which ultimately contributed to reduced adipogenesis. The piezoelectric hydrogel was implanted into estrogen-deficient rat models, resulting in increased bone growth and decreased adipogenic gene expression.

## 2 Results and discussion

### 2.1 Morphology and structure of MXene/PVDF/RSF hydrogel

Poly(vinylidene fluoride) (PVDF) is widely utilized for its unique piezoelectric properties. An electrospun PVDF fiber membrane with a high-voltage electrical coefficient was fabricated, featuring nanofibers with a uniform diameter of 0.75 μm that are intertwined to form a porous membrane network (Fig. 2A). The FT-IR spectra of the PVDF nanofiber membrane are shown (Fig. 2B), where there are more pronounced absorption peaks at 841, 1275, and 1434 cm^−1^, indicating a higher proportion of the β-crystalline phases in the structure after electrospinning [34]. In contrast, PVDF powder exhibited a peak at 763 cm^−1^ that corresponds to the α-phases of PVDF. The XRD pattern showed a narrow and intensified β-phase crystallization peak at 20.6° for the PVDF membrane, corresponding to the (110/200) crystal plane, while the PVDF powder displayed broader peaks at 18.4°, 20°, and 26.6° that correspond to the α-phase crystal planes (020), (110), and (021), respectively (Fig. 2C) [34]. The optimized β-phase content in the PVDF membranes exhibited the highest dipole moment, making them suitable for being the core of the piezoelectric hydrogel [21].

**Fig. 2:**
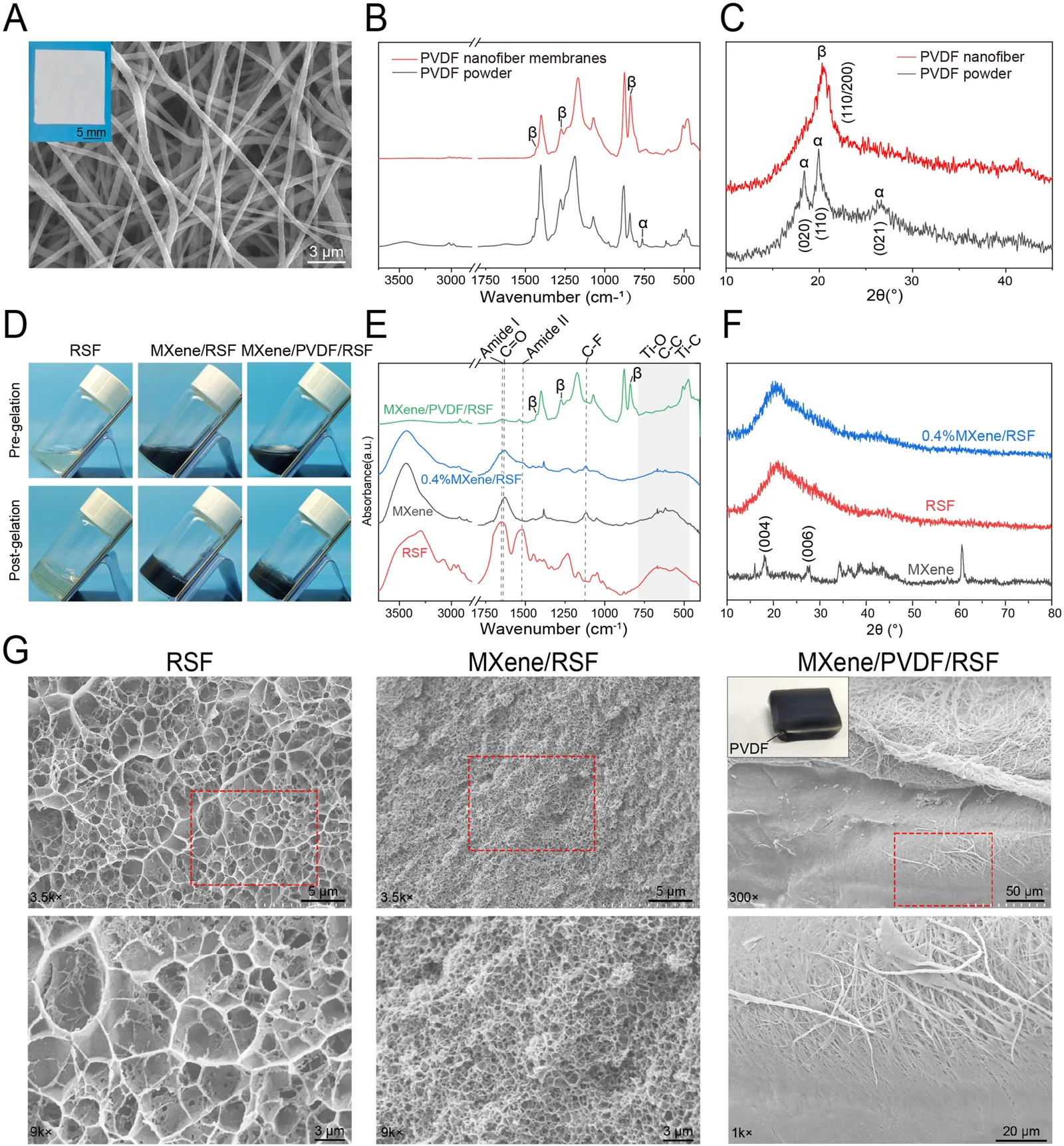
Fabrication of PVDF and piezoelectric hydrogel. (A) The as-made PVDF membrane (inset) and nanofibrotic structure were observed by SEM. (B-C) FTIR and XRD spectra of PVDF powder and electrospun membrane. (D) Pictures of before and after gelation of the RSF composite hydrogel. MXene was 4 mg/ml in each gel. (E-F) FTIR and XRD spectra of RSF composite hydrogel and MXene. (G) SEM images of RSF, RSF with MXene, and PVDF membranes sandwiched in MXene-RSF hydrogel.

The piezoelectric RSF/MXene-based hydrogel embedded with the electrospun PVDF membranes formed a sandwich structure and gelled (Fig. 2D). During electrospinning, PVDF is stretched to form more β-crystalline phases compared to electrospraying, and the piezoelectric performance can be adjusted by simply inserting more or fewer layers [35]. The fingerprint region of MXene at 1400 to 400 cm^−1^ shows bending vibration of Ti-O, C-C, and Ti-C stretching vibration peaks, which range between 650 and 350 cm^−1^ [36]. Additionally, the C-F peak of MXene was located at 1115 cm^−1^, whereas the C=O peak at 1623 cm^−1^ was strong enough to mask the amide I and amide II structure of RSF after compositing (Fig. 2E) [37]. The XRD pattern of MXene shows the crystal planes of (004) and (006) at 18° and 27.4°, respectively (Fig. 2F) [38]. The characteristic broad peak of silk II at 20.6° that corresponds to the antipolar, antiparallel β-sheet structure was maintained in the MXene/RSF composite, indicating that MXene did not disrupt the silk II [39], [40].

Typically, RSF exhibits a beehive-like morphology with larger pore sizes due to ice crystal expansion. Adding MXene resulted in a uniform, smaller pore size and a denser hydrogel structure, suggesting that the crystallization of ice was limited as MXene nanosheets bound the RSF fibrils [41]. After sandwiching, the electrospun PVDF maintained its fibrous structure between MXene/RSF hydrogels (Fig. 2G).

### 2.2 Mechanical and piezoelectric properties of MXene/PVDF/RSF hydrogel

The mechanical properties of MXene/RSF hydrogels were evaluated, revealing that modulus and compression at break were slightly higher compared to controls, as shown in the stress-strain curve (Fig. 3A). The average compressive strength of the hydrogels increased from 1.046 to 1.097 MPa after combining RSF with MXene. The average compressive strength of MXene/RSF hydrogels with 1, 2, and 3 layers of PVDF was 1.093, 1.087, and 1.089 MPa, respectively (Fig. 3B). The compressive moduli increased moderately from 1.116 to 1.306 MPa after adding MXene, attributed to the binding and consolidation of RSF fibrils. The addition of PVDF layers did not change the moduli of the hydrogel very much, ranging from 1.246 to 1.289 to 1.155 MPa for 1, 2, and 3 layers of PVDF membranes (Fig. 3B). The mechanical strength of MXene/PVDF/RSF hydrogel is two-fold higher than a few previous RSF-based hydrogels for treating normal and osteoporotic femoral defects, which have a compressive strength of around 500 kPa [42], [43], [44].

**Fig. 3:**
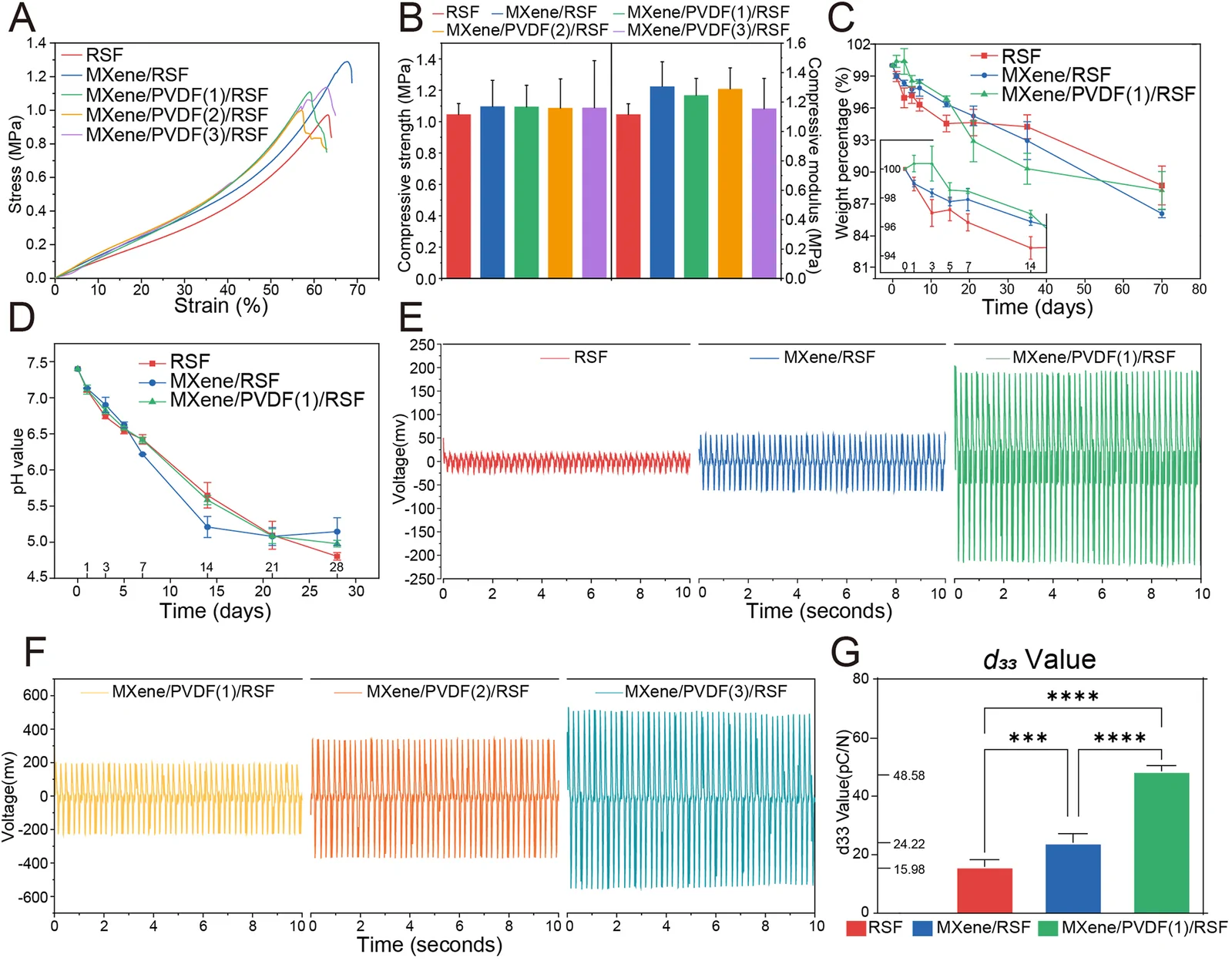
Mechanical properties, degradation, and piezoelectric properties of MXene/PVDF/RSF hydrogels. (A) Compressive stress-strain curves, (B) compressive strength, and moduli of hydrogels. (C) The weight loss of the entire degradation period, and the inset shows the first 14 days. (D) pH changes of 28 days. (E) The voltages were generated by RSF alone, MXene/RSF hydrogel, and MXene/PVDF(1)/RSF hydrogel. (F) The voltages generated by MXene/RSF hydrogels with one, two, or three layers of PVDF membranes. (G) *d*_*33*_ value of RSF alone, MXene/RSF hydrogel, and MXene/PVDF(1)/RSF hydrogel. *n* = 4, analyzed by ANOVA followed by Tukey's multiple comparisons test, *: *p* < 0.05, **: *p* < 0.01, ***: *p* < 0.001, ****: *p* < 0.0001.

The degradation behavior of RSF composite hydrogels with α-chymotrypsin was investigated, which involves the carboxyl groups located at the hydrophilic regions of RSF [45], [46]. During the first 14 days of degradation, the hydrogel made solely with RSF degraded faster than MXene/RSF and MXene/PVDF/RSF (Fig. 3C). As the SEM images show (Fig. 2G), adding MXene to RSF decreased the pore sizes of RSF fibrils, of which α-chymotrypsin hardly accessed the hydrophilic regions. After 35 days, faster weight loss was observed in MXene/PVDF/RSF with one layer of PVDF, as the crevices between PVDF and the MXene/RSF gel allow more surface area to contact α-chymotrypsin. However, the weight loss that occurred at 70 days was not significant. Thus, the sandwich structure did not significantly speed up degradation. In the meantime, MXene and PVDF did not affect the activity of α-chymotrypsin (Fig. 3C). Since the RSF is a protein biopolymer, enzymatic degradation through hydrolysis by serine 195 and histidine 57 of α-chymotrypsin involves attacking peptide bonds and aromatic side groups [47]. Degradative products and acids were released and slowly acidified the PBS solution, reaching a pH of about five after 28 days (Fig. 3D). The carboxyl-terminal side of aromatic or large hydrophobic amino acids such as glycine, alanine, tyrosine, threonine, tryptophan, phenylalanine, and methionine within the amorphous region of RSF was accessible to α-chymotrypsin, and the degradative silk fibroin peptides have isoelectric points of 4.0–6.8, determined by LC-MS/MS [48], [49]. Thus, these acidic degradative products are thought to decrease the pH of the buffer.

As PVDF membranes possess excellent piezoelectric properties after electrospinning, a self-powered hydrogel should generate piezoelectricity when being compressed. The voltages generated by the RSF hydrogel with MXene and PVDF membranes were measured, revealing an irregular electric potential (25.33 ± 1.086 mV) when the RSF hydrogel alone was compressed. The voltage waves became regular and steady at 53.99 ± 2.377 mV for MXene/RSF hydrogels due to the conductivity of MXene. The voltage increased significantly to about 198.6 ± 3.164 mV when an MXene/RSF hydrogel with one layer of PVDF membrane was measured (Fig. 3E), and the current was about 2500 nA (Fig. S1). Moreover, the voltages increased to 325.5 ± 33.41 mV and then to 491.7 ± 5.681 mV when PVDF membranes were one to two layers and then three layers (Fig. 3F). The piezoelectric coefficient (*d*_*33*_) rose from 15.98 pC/N with a standard deviation (SD) of 2.358 pC/N for RSF alone to 24.22 pC/N with an SD of 2.992 pC/N and then to 48.58 pC/N with an SD of 1.893 pC/N (Fig. 3G). Thus, inserting layers of PVDF membranes can meet the requirements of various situations. According to Tu et al., charges accumulate on the MXene even at low concentration in a composite when an electrical field is applied, as explained by the microscopic dipole model [50]. Thus, when the electric field was constructed by PVDF membranes in response to stress, the MXene presented interfacial polarization and increased the *d*_33_ to 63.3 pC/N. By combining the adjustable high-piezoelectric PVDF membranes with the conductive capabilities of MXene, which enhance the piezoelectric effect, the MXene/PVDF/RSF hydrogel is suitable for evaluating biological potential.

### 2.3 Piezoelectricity stimulates osteogenesis and reduces adipogenesis of inflammatory bone cells

The viability of MC3T3 was assessed in the extract of hydrogels containing different layers of PVDF membranes. The number of PVDF membranes did not significantly affect cell viability, as most of the cells showed green fluorescence, while red fluorescence was sparse. Additionally, although the cells grew more confluent in the wells without PVDF membrane extracts, the difference was not significant (Fig. S2). Therefore, three layers of PVDF membranes were optimal and used in subsequent experiments.

For studying the biological response to piezoelectricity, we assembled a compression device composed of plastic sheets, sticks, solenoids, relays, and a power line, and the size was compact to fit in a cell incubator. (Fig. 4A). The compression device generated up to 8 N of force repetitively (Fig. 4A inset). The maximal internal force applied to the distal femur in a rat is approximately 7 N using modeling by micro-CT scan. Thus, the force generated by the compression device is comparable to muscle contraction during the gait cycle [51]. The frequency was set to 2 times per minute and continuously compressed the MXene/PVDF/RSF hydrogel, where cells were seeded on the culture dishes in the middle of the circular, ring-shaped hydrogel placed within, mimicking muscle contraction that induces the piezoelectric effect. The piezoelectricity generated by MXene/PVDF(1)/RSF induced calcium flux in MC3T3 cells after 60 s of stimulation, indicating responsiveness to piezoelectric stimulation. The fluorescence was much dimmer when cells were preincubated with tetrodotoxin, which is a calcium channel blocker that inhibits piezoelectric cellular response [52] (Fig. 4B and C).

**Fig. 4:**
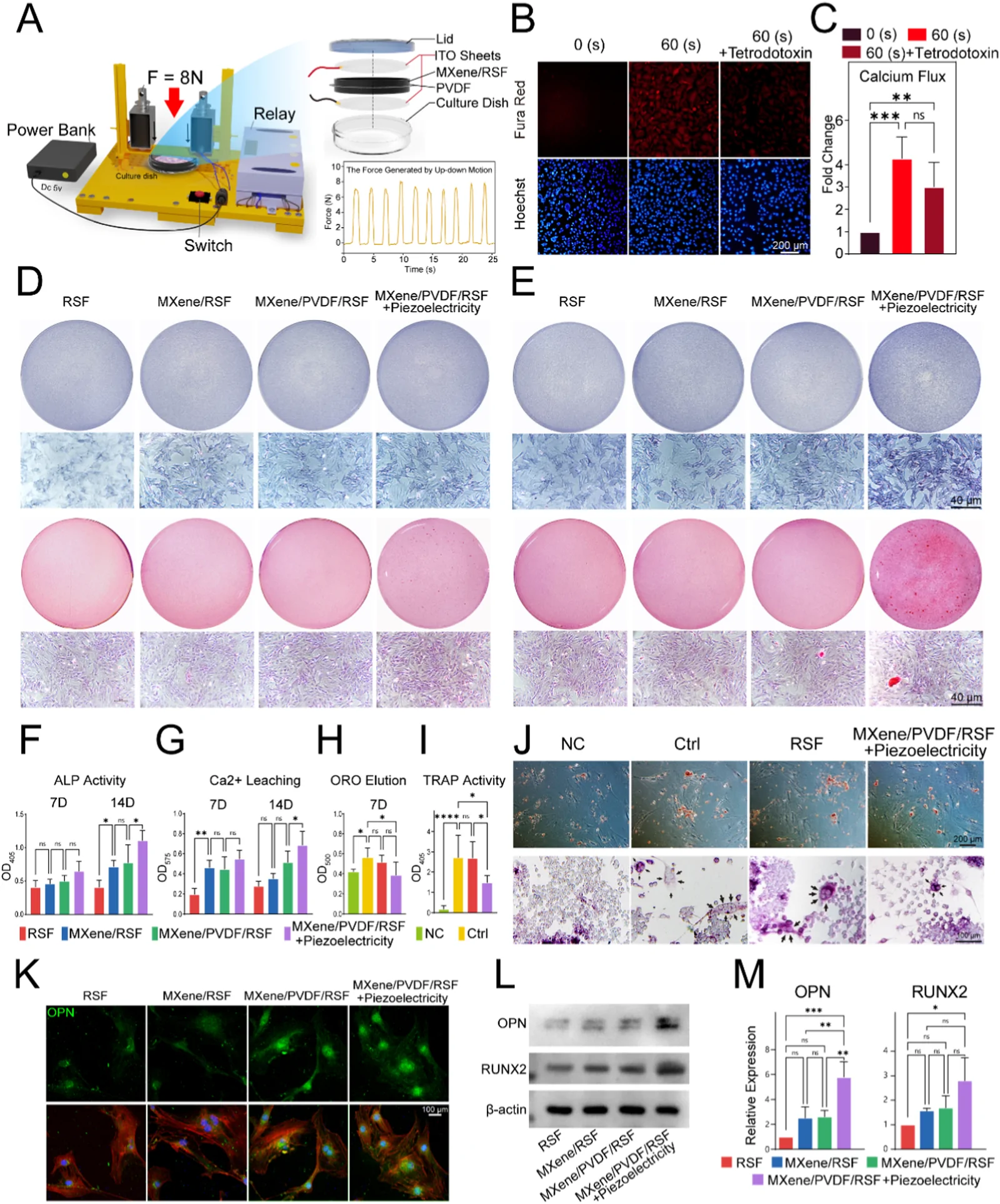
Osteogenesis-adipogenesis of inflammatory bone cells was balanced by piezoelectricity. (A) A ring-shaped MXene/PVDF/RSF hydrogel was assembled and placed in a culture dish, which was then placed in the proper position to receive compressive stress from a handmade compression device, generating a repetitive series of downward forces up to 8 N. (B) Calcium flux in MC3T3 cells was indicated by Fura Red fluorescence when stimulated by piezoelectricity. (C) Quantitative analysis of calcium flux induced by piezoelectric MXene/PVDF(1)/RSF hydrogel, *n* = 5. (D, E) ALP staining and ARS staining of rBMSCs stimulated by piezoelectricity from MXene/PVDF/RSF hydrogels, (F) ALP activity assay, (G) calcium leaching assay at 7 and 14 days, *n* = 5. (H) Lipid droplets were stained with Oil Red O and quantified by absorbance at 500 nm, *n* = 6. (I) Osteoclast activity was quantified with a TRAP assay and measured by absorbance at 405 nm, *n* = 6. (J) Adipogenesis and osteoclastogenesis were reduced by piezoelectricity. The lipids were visualized by Oil Red O staining at 7 days. The osteoclasts were stained with TRAP staining, *n* = 6. Black arrows indicate osteoclasts. (K) Immunostaining of OPN in MC3T3 cells merged with β-actin and the nucleus. (L) Expression of osteogenic markers OPN and RUNX2, and (M) relative expression. *n* = 3, analyzed by ANOVA followed by Tukey's multiple comparisons test, *: *p* < 0.05, **: *p* < 0.01, ***: *p* < 0.001.

As a key driver of inflammation, elevated TNF-α levels were also found in ovariectomized rats and postmenopausal women. [53], [54]. Thus, we mimicked the inflammatory environment in defects by adding TNF-α to the culture medium. First, osteogenesis and cell viability were evaluated by ALP activity assay, calcium deposition assay, and cell viability assay, showing minimal effects on bone cells (Fig. S3). Next, osteogenesis affected by the piezoelectricity was evaluated. After 7 days of piezoelectric stimulation, ALP staining was moderately darker than that of the hydrogel without compression, and the calcium deposits were not significantly different (Fig. 4D, F, G). After 14 days of piezoelectric stimulation, the activity of ALP was more vigorous than that of the others without stimulation. Calcium deposits were diffusely distributed on the BMSCs, compared to the unevenly stained cells without stimulation (Fig. 4E, F, G). Furthermore, the adipogenic differentiation and osteoclastogenesis of rBMSCs were induced with adipogenic culture medium or M-CSF and RANKL, respectively (Fig. 4J, upper panel). The lipid droplets stained by Oil Red O were more abundant in the control cells and cells cultured in RSF hydrogels. In contrast, fewer and smaller lipid droplets were found in the rBMSCs stimulated by piezoelectricity and were significantly reduced compared to control cells (Fig. 4H). As for osteoclastogenesis, without M-CSF and RANKL, no osteoclasts were found in NC. In Ctrl, macrophages fused and became much larger. After TRAP staining, 2-7 nuclei were visible in single cells due to cell fusion. In RSF, cells were co-cultured with RSF hydrogel, and fused large osteoclasts with multiple nuclei were also observed. When cells were co-cultured and stimulated by piezoelectric stimulation, fewer large osteoclasts with more than one nucleus were found (Fig. 4J, lower panel). Osteoclast activity was confirmed by quantifying tartrate-resistant acid phosphatase (TRAP) staining (Fig. 4I), which breaks down phosphate groups in bone and causes bone loss. Thus, piezoelectric stimulation generated by MXene/PVDF/RSF hydrogel effectively reduced osteoclast formation. The expression of osteopontin in the MC3T3 cells was also examined, with brighter fluorescence observed in the cytoplasm of the cells stimulated by piezoelectricity (Fig. 4K). Western blot analysis confirmed higher expression of osteopontin and RUNX2, a transcription factor critical for osteoblast differentiation, in the group stimulated by piezoelectricity (Fig. 4L and M). These results indicate that piezoelectricity can effectively rebalance bone-fat balance by enhancing osteoblast differentiation, reducing adipogenesis, and suppressing osteoclastogenesis at the same time.

### 2.4 Genes of glycerophospholipid metabolism were altered in inflammatory bone cells in response to piezoelectricity

Bone-fat imbalance is a phenomenon observed in several metabolic disorders, such as diabetes, which is characterized by increased adipose tissue and aberrant bone mineralization [55], [56], [57]. Thus, we delve into exploring the transcription and metabolism changes in response to piezoelectric stimulation. We stimulated MC3T3 cells by subjecting them to piezoelectricity, then extracted the mRNA and metabolites for multi-omics analysis. First, we compare the mRNA level and metabolite abundance between cells in MXene/PVDF/RSF without TNF-α and cells cultured in MXene/PVDF/RSF with TNF-α (Fig. 5A). Differentially expressed mRNAs were enriched in 88 pathways, while different metabolites were enriched in 62 pathways. Among these, 14 pathways were activated and contributed to the downstream metabolism (Fig. 5B). Next, we compared the cells cultured with piezoelectricity generated by MXene/PVDF/RSF and the cells without piezoelectric stimulation. Differentially expressed genes were enriched in 79 pathways, and the different metabolites were enriched in 46 pathways. Among these pathways, 8 were activated by differentially expressed genes and contributed to metabolism (Fig. 5C and D). Notably, glycerophospholipid metabolism was activated throughout the three groups, as revealed by combining transcriptomics and metabolomics analyses (Fig. 5E and F). Among these genes, the expression of those dominating downstream glycerophospholipid degradation was initially altered by TNF-α but subsequently reversed by piezoelectricity, indicating that these target genes regulate this metabolic shift (Fig. 5G). To verify the role Pla2g4a plays in lipid degradation, we repeated RT-qPCR experiments after piezoelectric stimulation. Pla2g4a expression following piezoelectric stimulation was 5.7-fold higher than that in cells without stimulation. When cells were treated with siRNA, Pla2g4a fell to 0.29-fold, showing about 70% efficiency (Fig. 5H). The encoded protein cPLA2 expression was studied by Western blot, and piezoelectric stimulation induced cPLA2 expression threefold compared with cells without stimulation. After siRNA knockdown, cPLA2 dropped to about 0.53-fold (Fig. 5I and J). The adipogenesis reduction was visualized by Sudan black B staining, which stained glycerophospholipids profusely in the rBMSCs without piezoelectricity. Compared to the fewer black stains in the rBMSCs, the adipogenesis-reducing effect of piezoelectric stimulation was reversed after silencing Pla2g4a expression by siRNA, indicating that lipid degradation via Pla2g4a was induced by piezoelectricity (Fig. 5K and L). Thus, cPLA2 expression should be responsible for the lipid droplet results by Sudan Black B staining. cPLA2 is the protein encoded by Pla2g4a; it catalyzes membrane lipid hydrolysis and releases arachidonic acid for PGE_2_ production [58]. Studies reported that initial PGE_2_ production from COX-2 facilitates recruitment of osteoblasts [59]. Thus, piezoelectric stimulation-induced cPLA2 expression may promote PGE_2_ release from adipogenic cells, potentially contributing to bone regeneration. The limitation is that Pla2g4a was initially explored using the pre-osteoblast MC3T3 cell line; the gene and metabolite profile could not directly recapitulate the molecular processes associated with osteogenic–adipogenic lineage commitment of BMSCs. Nevertheless, the multi-omics results still provide mechanistic insights into the glycerophospholipid metabolism in response to piezoelectric stimulation. To complement the multi-omics findings, we performed the adipogenic differentiation experiments using rBMSCs and confirmed the role cPLA2 plays in adipogenesis. Thus, cPLA2 may represent an important regulatory node linking glycerophospholipid metabolism with cellular differentiation responses across different cell types [60].

**Fig. 5:**
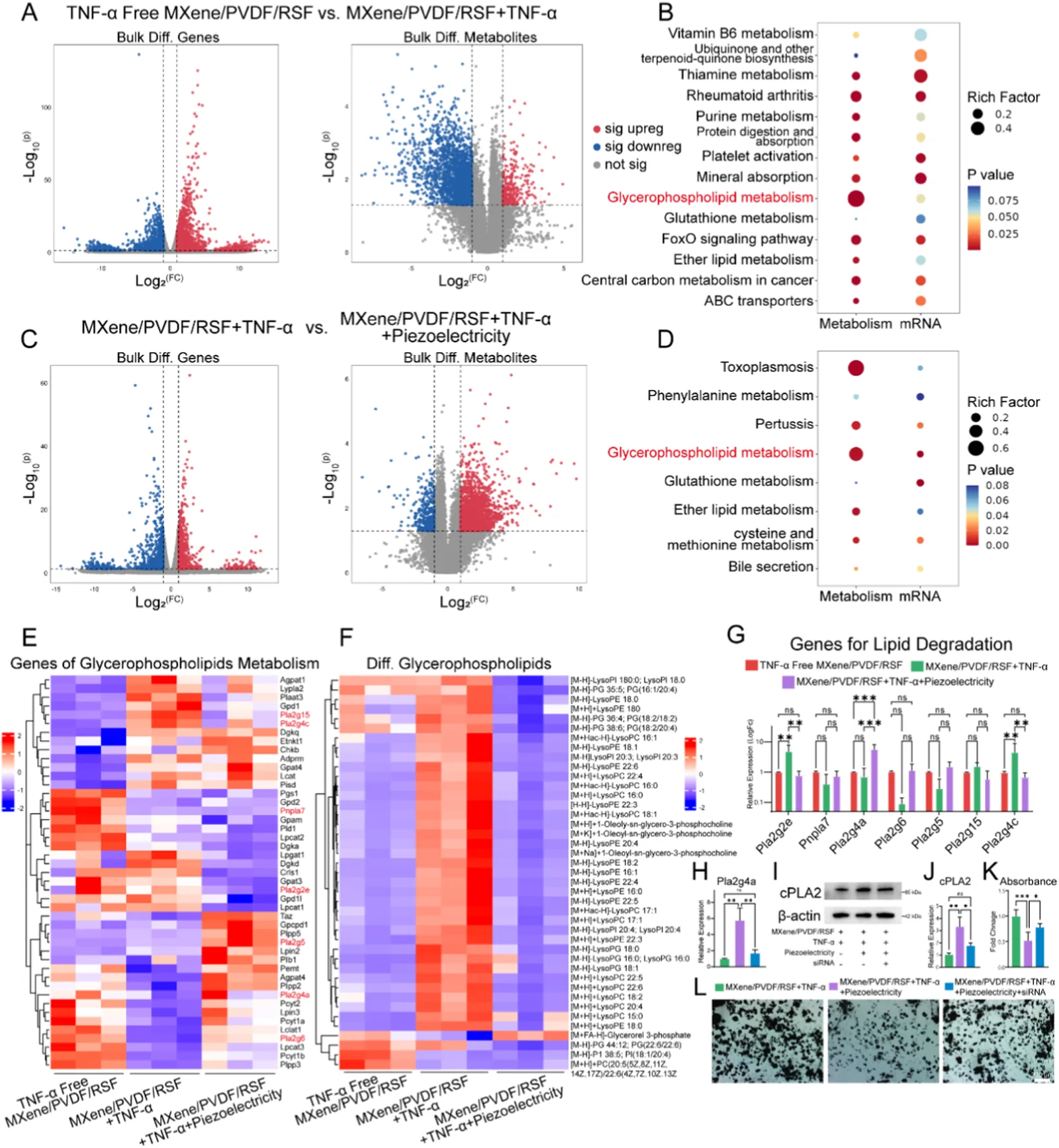
Multi-omics analysis of gene and metabolite expression induced by piezoelectricity. (A) Volcano plots of gene and metabolite expression from MC3T3 cells in MXene/PVDF/RSF without TNF-α vs. cells cultured in MXene/PVDF/RSF hydrogels with TNF-α, and (B) KEGG enrichment of differentially expressed mRNA and metabolites. (C) Volcano plots of gene and metabolite expression from MC3T3 cells cultured in MXene/PVDF/RSF hydrogels without piezoelectricity vs. cells stimulated by piezoelectricity generated from MXene/PVDF/RSF hydrogels, and (D) KEGG enrichment of differentially expressed mRNA and metabolites. (E, F) Heatmaps of genes related to glycerophospholipids and glycerophospholipid species between TNF-α-free MXene/PVDF/RSF, MXene/PVDF/RSF + TNF-α, and MXene/PVDF/RSF + TNF-α + Piezoelectricity. (G) Gene expression related to lipid degradation was verified by RT-qPCR. *n* = 3, analyzed by ANOVA followed by Tukey's multiple comparisons test. (H) Pla2g4a expression in rBMSCs treated without piezoelectricity, with piezoelectricity, and with piezoelectricity plus siRNA. (I) cPLA2 expression results from the Western blot and (J) quantified as relative expression. *n* = 3, analyzed by ANOVA followed by Tukey's multiple comparisons test. (K) Quantification of Sudan Black B staining by fold change of absorbance at 600 nm. *n* = 5, analyzed by ANOVA followed by Tukey's multiple comparisons test. (L) Representative pictures of Sudan Black B staining of lipids. *: p < 0.05, **: p < 0.01, ***: p < 0.001.

### 2.5 Bone-fat imbalance is a key feature in the OVX rat femurs

To investigate the possible bone disorder of the inflammatory environment in osteoporosis in vivo, an OVX (ovariectomy-induced estrogen deficiency model) rat model was established by resecting the bilateral ovaries, as estrogen withdrawal leads to an inflammatory bone environment. After three months, the OVX rats gained significantly more weight than the rats without ovary removal, suggesting altered lipid metabolism. Micro-CT scanning found that bone volume and bone surface decreased in the femurs of OVX rats (Fig. 6A, D). The major change were in the trabeculae features in subchondral bone and beneath the epiphyseal plate, which changed significantly with increased trabeculae thickness, decreased number, and increased separation (Fig. 6E). Next, the bone mineral density (BMD) assessed by DXA (dual-energy X-ray absorptiometry) moderately decreased in the spine vertebrae but not significantly. However, femoral BMD decreased significantly, indicating the femur is the primary target of bone loss (Fig. 6F). The femur composition was characterized by an increased fat-to-total weight ratio, suggesting the lost bone was replaced by adipose tissue in the femoral bone marrow (Fig. 6G). H&E staining shows that the trabeculae in OVX were fewer, and the spaces in between were replaced by oval-shaped adipocytes, of which the lipids inside were removed during sample preparation and left unstained. Morphological analysis indicated that the number of bone marrow adipocytes increased, whereas their size did not (Fig. 6B, H). Immunostaining of osteoinductive BMP2 and adipoinductive PPARγ shows that the relative red to green intensity decreased significantly in the subchondral and epiphyseal plate areas (Fig. 6C, I).

**Fig. 6:**
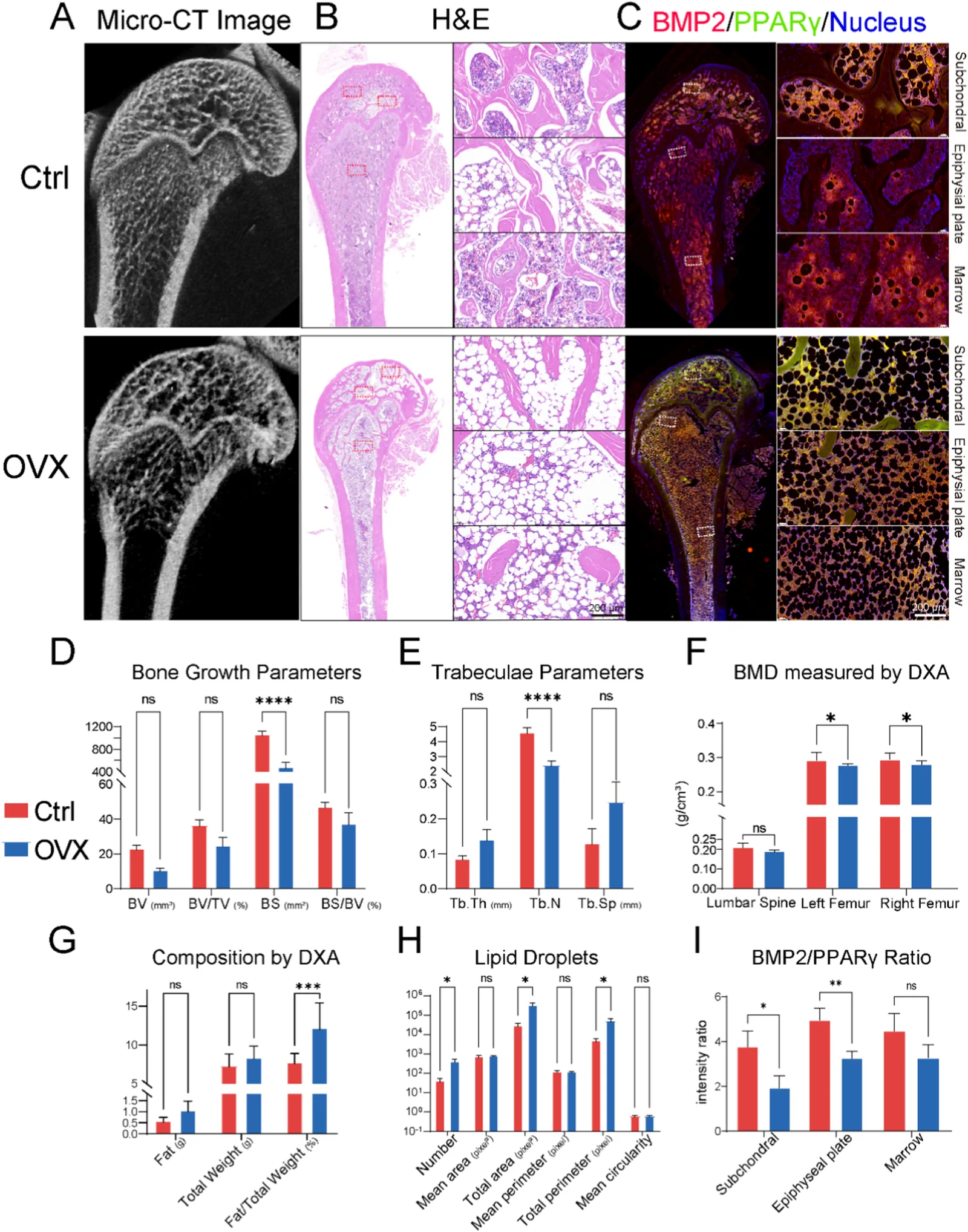
Structural and adipogenic changes in estrogen-deficient femur. (A) Sagittal planes of micro-CT images and (B) H&E staining of control and estrogen-deficient distal femurs. (C) Images of BMP2 and PPARγ expression in control and estrogen-deficient femurs. (D) Bone growth, morphology, and (E) trabeculae morphology were assessed by micro-CT. *n* = 6, analyzed by ANOVA followed by Tukey's test. (F) BMD of the lumbar spine and distal femurs was assessed by DXA, and (G) composition regarding fat and total weight of the ROI. *n* = 5, analyzed by ANOVA followed by Tukey's test. (H) Morphology of lipid droplets and (I) relative intensity ratio of BMP2 to PPARγ in control and estrogen-deficient femurs. *n* = 3, analyzed by Student's t-test. *: *p* < 0.05, **: *p* < 0.01, ***: *p* < 0.001.

### 2.6 MXene/PVDF/RSF hydrogel restores bone-fat balance in the OVX femoral defect

Before creating defects in the femoral condyles, we measured the kicking force generated by a single hindlimb by modifying the method developed by Zheng et al. [60]. The average and standard deviation of kicking force were 6.688 ± 0.5650 N, which is comparable to the force used for in vitro experiments. To investigate whether piezoelectricity generated by MXene/PVDF/RSF hydrogel is helpful in bone healing, a 3 mm wide femoral defect was created in OVX rats, into which MXene/PVDF/RSF hydrogel was implanted (Fig. 7A). After 6 weeks, the bones grew around No Implant, RSF, MXene/RSF, and PVDF/RSF hydrogels, which were thinner than the piezoelectric MXene/PVDF/RSF hydrogel. The morphological analysis shows that without combining PVDF membranes and electrically conductive MXene, the BV/TV and BMD did not show a comparable increase to the MXene/PVDF/RSF scaffold at 6W. Although trabeculae parameters increased moderately, bone regeneration stimulated by piezoelectricity was mostly improved in BV/TV (Fig. S5A and B). The defects in the No Implant were left vacant, and bone grew little from the fringe of the defect after 12 weeks, which mineralized incompletely with lower BMD in the defect. In contrast, a thin layer of bone grew on the surface of the RSF hydrogel after 12 weeks, suggesting that the RSF hydrogel supported the defect from collapse and bone grew vigorously at the hydrogel-bone interface. The incorporation of MXene and PVDF rendered the RSF hydrogel responsive to the deformation of bone due to muscle contraction and simultaneously generated piezoelectricity, and the BV/TV was significantly higher than all the other hydrogels (Fig. 7B). The trabeculae thickness (Tb. Th) was higher in the defect stimulated by piezoelectricity, suggesting the deterioration induced by the inflammatory environment was alleviated (Fig. 7B). The piezoelectricity stimulated the bone around the defect in addition to supporting the bone structure, leading to thicker bone growing around the MXene/PVDF/RSF hydrogel.

**Fig. 7:**
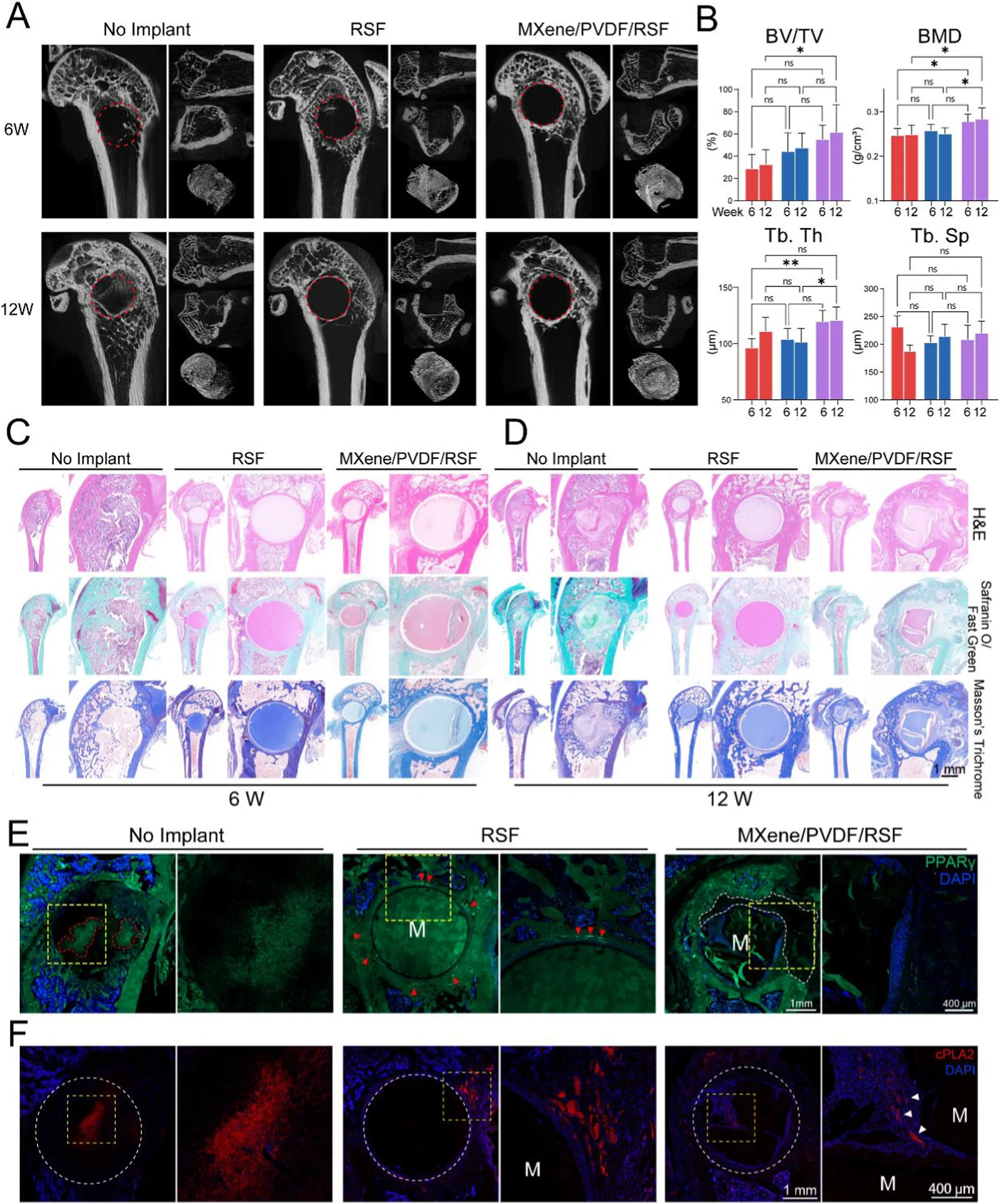
(A) Sagittal planes, coronal planes, transverse planes, and ROI of the distal femurs. (B) Bone volume to total volume, BMD, trabeculae thickness, and trabeculae separation measured by micro-CT. (C, D) H&E staining, Safranin O/Fast green staining, and Masson's trichrome staining of defects at 6 and 12 weeks. (E) Immunostaining of PPARγ in green and the nucleus in blue of defects at 12 weeks. The red dashed line and triangles indicate expression of PPARγ, the white dashed line indicates the area without PPARγ expression, and M represents hydrogels. (F) Immunostaining of cPLA2 in red and the nucleus of defects at 12 weeks. The white triangles indicate cPLA2. The white dashed circles indicate a defect. n = 6, analyzed by ANOVA followed by Tukey's test, *: p < 0.05, **: p < 0.01, ***: p < 0.001.

After 6 weeks, we found that the defect in the No Implant group was mainly filled with mesenchymal tissue, within which little immature bone grew. The trabeculae did not grow in the defects, and only a few collagen fibers were present instead. Numerous trabeculae-like bones grew around the defect over time. However, the newly formed bone did not coalesce into normal bone structures in the epiphysis. When the defects were filled with RSF hydrogel, a layer of bone grew along the surface and surrounded the RSF hydrogel. The layer of bone became thicker and connected with trabeculae nearby. Ethanol-treated RSF hydrogel contains more β-sheet structures and contributes to a relatively long degradation time (Fig. 3C), providing a platform for the bone to grow. With only PVDF or MXene, the hydrogel could not effectively produce piezoelectric stimulation, and the morphological observations were consistent with micro-CT results, showing a thinner layer of collagen around MXene/RSF and PVDF/RSF hydrogels (Fig. S5C). A layer of bone also grew around the defect implanted with MXene/PVDF/RSF hydrogel, which was much thicker than all the other groups (Fig. 7C). The defect in the No Implant group was still filled with disorganized mesenchyme, with only a trace of collagen fiber at 12 weeks. In the RSF group, the collagen fibers filled around RSF grafts but did not fully mineralize according to micro-CT analysis. The size of MXene/PVDF/RSF grafts became smaller, showing signs of early degradation, and the gaps were filled with mesenchyme and collagen fibers, which mineralized and matured into solid bone (Fig. 7D). With a lower modulus (Fig. 3B), the hydrogel degraded and changed shape while being compressed and generating piezoelectricity.

As for adipogenetic markers, PPARγ was highly expressed in the mesenchymal tissue that formed within the defects of No Implant (red dashed line) and moderately expressed in the newly grown tissue in close contact with RSF grafts (red triangle) but scarcely expressed in an area close to MXene/PVDF/RSF (white dashed line) (Fig. 7E), suggesting adipogenesis was reduced by the piezoelectricity from the MXene/PVDF/RSF. We also found that cPLA2 expression, encoded by Pla2g4a, was limited to the center of the ingrowth in the defect without implants or was restricted to an area when RSF was implanted. When the MXene/PVDF/RSF was implanted, the cPLA2 was expressed in the newly grown tissue where the implant was degraded and the piezoelectricity was generated (Fig. 7F).

## 3 Conclusion

In summary, we successfully achieved a tunable piezoelectric MXene/PVDF/RSF hydrogel for rehabilitation after bone defect implantation by optimizing the β-phase and layers of the PVDF membrane. The piezoelectricity effectively stimulated osteogenesis and reduced adipogenesis of inflammatory bone cells by targeting glycerophospholipid metabolism. Bone healing in the femoral defect affected by an inflammatory environment due to estrogen deficiency was enhanced by piezoelectric MXene/PVDF/RSF hydrogel, and the PPARγ expression, which is remarkable for adipogenesis, was reduced. Such a protein hydrogel that converts stress from muscle contraction into electrical stimulation is promising for treating various bone defects exacerbated by inflammation due to endocrine disorders.

## 4 Materials and methods

### 4.1 Preparation of silk fibroin

Natural mulberry cocoons were degummed by immersion in 5% sodium carbonate solution and dried. The degummed silk was dissolved with a 9.7 mol/L LiBr solution in a water bath at 60°C at a mass ratio of 1:10 and heated for 1 h. The RSF solution was cooled to room temperature and then dialyzed in a 3500 MWCO dialysis bag in deionized water for 72 h, during which the deionized water was changed every 8 h. This was followed by PEG concentration to obtain a 10% RSF solution.

### 4.2 Preparation of PVDF electrospun membranes and MXene/RSF hydrogel sandwiched with PVDF membranes

PVDF (99.9%, MW = 400,000, bioreagent grade, Shanghai Yuanye Bio-Technology Co., Ltd., w/v = 130 mg/ml) was dissolved in a mixture of dimethyl sulfoxide and acetone (DMSO/AC = 2/1). To achieve uniform fiber morphology, the PVDF electrospinning parameters were set at a flow rate of 1 mL/h, a voltage of 25 kV, and a spinning distance of 13 cm. This process was carried out in an environment maintained at 20-30°C and 40-60% relative humidity, yielding fiber membranes with a final thickness of 0.2-0.3 mm. The spun membrane was dried in a vacuum oven at 50°C for 24 h to remove the residual solvent.

Firstly, a 10% RSF solution and MXene (Ti_3_C_2_Tx; purity: 56-68 wt%; XFNANO; 4 mg/ml; thickness: 100-200 nm; diameter: 2-10 μm; element analysis is in Table S1) were mixed, and then 900 U/ml horseradish peroxidase (HRP) and 0.5% H_2_O_2_ were added. Secondly, one-third of the MXene/RSF solution was poured into a mold, then 1, 2, or 3 layers of PVDF membranes were consecutively stacked on the solution surface. When stacking PVDF membranes, one-third of the MXene/RSF solution was dripped in between them to ensure no air bubbles were trapped and to facilitate effective electron transfer between the PVDF membranes. The remaining one-third of the MXene/RSF solution was poured for the upper half of the hydrogel composite. Thirdly, the mold was carefully disassembled after gelling, and the hydrogel composite was soaked in a 75% ethanol solution for 24 h to induce β-sheet formation.

### 4.3 Morphology, degradation, and piezoelectricity

Photographs of RSF hydrogel, MXene/RSF hydrogel, and PVDF/MXene/RSF hydrogel were taken before and after gel formation using a digital camera. The microstructures of PVDF fibers, RSF hydrogel, and MXene/RSF hydrogel were characterized using a Hitachi S-3400 at 15 kV. Hydrogel structures were analyzed using a Fourier infrared spectrometer (Nicolet iS50, USA) and an X-ray diffractometer (D/Max2550, Japan).

Each set of samples was immersed in 10 mL of PBS solution with 240 U/mL α-chymotrypsin, then placed in a shaker and kept at 37°C to simulate degradation [45]. At each time point, the samples were transferred into fresh α-chymotrypsin solution to maintain enzymatic activity. The hydrogels were thoroughly washed with deionized water, and then the surface was dried with filter paper and weighed. For the pH experiment, each set of samples was immersed in 10 mL of PBS (pH = 7.4) buffer (Servicebio, G4202), and the pH value of the solution was measured at predetermined time points using a pH meter.

Piezoelectricity generated in response to stress was recorded by attaching copper leads to ITO sheets. The leads were placed on the upper and lower surfaces of the hydrogels and secured by polyimide tape. The digital multimeter KEITHLEY DMM6500 linkage was set to DC mode for recording voltages and currents. Piezoelectric hydrogel samples were placed in a quasi-static *d*_33_ m (YINGAISHI, ZJ-3A+), and d_33_ coefficients were measured for piezoelectric efficiency.

### 4.4 Characterization of MXene/PVDF/RSF hydrogel

The compression test was analyzed by a universal testing machine (SANS CMT 2503, USA) at a 10 mm/min strain rate. The stress-strain curves were plotted using OriginLab, and the initial 5% of compression was used for calculating compressive moduli. The force generated by the compression device was measured by tying a string from the moving plastic part that compresses the MXene/PVDF/RSF hydrogel to a clamp module of a universal testing machine.

Piezoelectric hydrogels were freeze-dried at 5 Pa and −37°C for 2 days. Dried samples were ground into powder for FT-IR analysis (ThermoFisher, Nicolet 6700). The absorbance wavelength between 3700 and 450 cm^−1^ was recorded and analyzed by OriginLab.

Hydrogels were freeze-dried, and diffraction peaks were analyzed by X-ray from a line source of Cu Kα at 40 kV, 100 mA (18 KW/D/max 2550VB/PC, Japan), with an incident light wavelength of 1.54 Å and a scanning speed of 3°/min.

### 4.5 BMSC culture and in situ piezoelectricity generation

To stimulate BMSCs with the piezoelectric current generated by the MXene/PVDF/RSF hydrogel in its original place, we handmade a small portable compression device to compress the hydrogel placed inside the culture dish with seeded cells. Briefly, the device comprises a platform, two columns, a plastic sheet attached to solenoids to push a lid, a tunable relay, and a power outlet with a power line. The plastic sheet pushes the lid of the culture dish downward at the set time. A circular MXene/PVDF/RSF hydrogel ring, slightly taller (1-2 mm) than the culture dishes, with 3.4 cm and 2.5 cm outer and inner diameters, was fabricated using a polytetrafluoroethylene mold and was placed inside a culture dish. The frequency was set to 2 times per minute in continuous mode. The compressive force was 8 N with 200 mV of voltage, 30% strain of hydrogels, and a 40% duty cycle. BMSCs were harvested from the femurs of 8-week-old SD rats euthanized by CO_2_ and cultured in DMEM supplemented with 10% fetal bovine serum (Thermofisher) at 37°C with 5% CO_2_. The BMSCs at a density of 5 × 10^5^/ml were seeded on the bottom of a culture dish, where there is a vacant area, and the culture medium in the culture dish facilitates electrical current transmission.

### 4.6 Cell viability, biocompatibility, and calcium flux

MC3T3 cells were seeded in 6-well plates at a density of 5 × 10^5^/ml and cultured with MXene/PVDF/RSF hydrogel extracts with one, two, or three layers of PVDF membranes. The viability was assessed on days 1, 3, and 5 by the Live/Dead Assay (Yeasen, 40747ES76). Calcein AM and PI were added to the culture medium, and the cells were incubated at 37°C for 30 min. The green and red fluorescence were observed using the corresponding filter sets, and pictures were taken with a digital camera (Olympus DP71). The live-to-dead ratio was calculated as the number of live cells divided by the number of dead cells and expressed as a percentage. Calcium flux in MC3T3 cells stimulated by piezoelectricity was indicated by loading Fura Red (Thermofisher). The MC3T3 cells were stimulated by piezoelectricity generated by MXene/PVDF/RSF for 60 s or alternatively preincubated with tetrodotoxin (100 nM, Sigma-Aldrich) and then observed under a microscope (Olympus DP71). The calcium flux was quantitatively analyzed using ImageJ 1.54f by measuring the average fluorescence intensity of five sets of samples and normalizing to the number of nuclei.

### 4.7 RT-qPCR

Cell RNA was extracted using the TRIzol agent and reverse transcribed to cDNA (Yeasen, 14601ES03) and stored at −20°C. RT-qPCR was performed by mixing cDNA, primers, and SYBR Green Master Mix (Yeasen, 11202ES08). The reaction was run by QuanStudio 5 (Thermo Fisher) and semi-quantified by the delta CT method. The forward and reverse primer sequences are

Pla2g2e: 5′-GACACAGACACACACACAC-3′, 5′-TGACTCCAGACATTTCCCC-3’.

Pnpl7: 5′-CGTGTTTTCCAACGACCACC-3′, 5′-TCTGCTAGTGCCCTGAGGAT-3’.

Pla2g5: 5′-TCATCTGCGAACACGACTCC-3′, 5′-TCCATCCCCGAGAGCATACA-3’.

Pla2g15: 5′-GCCACCACCTTGGCTTATCT-3′, 5′-GGGAAGCTCTAAGCAACCGT-3’.

Pla2g4c: 5′-GAGTGCTGCCCTATCCCATC-3′, 5′-GGTCCCAAACTAGACCCGTG-3’.

Pla2g6: 5′-CAGACCAACTGGTATGGCGT-3′, 5′-CCTGTCCCCAGAGAAACGAC-3’.

Pla2g4a: 5′-TGCTCACTCTCCTTTCCTC-3′, 5′-ACACACACACACACACAC-3’.

### 4.8 Osteogenesis by ALP, ARS staining, immunofluorescence, and Western blot; osteoclastogenesis by TRAP staining; adipogenesis by Oil Red O staining and Sudan black B staining

BMSCs were cultured in 3.5 cm culture dishes at a density of 5 × 10^5^/ml with Osteogenic Differentiation Medium (Pricella, PD-033) supplemented with TNF-α at 50 ng/ml to mimic an inflammatory environment (Abclonal, RP01322) until confluence. MXene/PVDF/RSF hydrogels were sterilized with 75% alcohol and immersed in PBS to eliminate alcohol. After continuous piezoelectric stimulation by MXene/PVDF(3)/RSF hydrogel for 7 and 14 days, cells were fixed with 4% paraformaldehyde or 75% alcohol and stained with ALP staining solution (Beyotime, C3206) or ARS staining solution (Beyotime, C0148S) for 1 h. Images were taken with a digital camera and a microscope (Olympus DP71). Macrophages were induced by M-CSF (Abclonal, RP01216S), followed by RANKL (Abclonal, RP02134) for osteoclast induction. After piezoelectric stimulation, osteoclasts were fixed with 4% paraformaldehyde and stained by a TRAP staining kit (Servicebio, G1050-50T) and found under a microscope (Olympus DP71). The TRAP activity was measured by a TRAP activity assay (Beyotime, P0332) following the instructions by adding para-nitrophenyl phosphate and *p*-nitrophenol, and absorbance at 405 nm was recorded by a plate reader (Molecular Devices, SpectraMax iD3). The BMSCs were passaged and induced adipogenesis using adipogenic differentiation medium (Pricella, PD-010) with TNF-α (Abclonal, RP01322) and subjected to continuous piezoelectric stimulation by MXene/PVDF(3)/RSF hydrogel for 2 weeks. For the Pla2g4a silencing experiment, BMSCs were induced to undergo adipogenesis with adipogenic differentiation medium, and siRNA (RiboBio company) for Pla2g4a was diluted in Lipofectamine 3000 transfection reagent (Thermofisher), mixed with Opti-MEM, and then added to BMSCs. The transfection was performed every three days. After 14 days, cells were fixed with 10% formalin and rinsed. The lipids were stained with Oil Red O (Beyotime, C0158S), and images were taken with a microscope (Olympus DP71). The ORO stain was eluted by adding 100% 2-propanol to each well; the 2-propanol with stain was transferred to another plate, and the absorbance at OD 500 nm was measured by a plate reader (Molecular Devices, SpectraMax iD3). Sudan black B (Beyotime Y2698321) powder was dissolved in 70% ethanol, then added to the fixed cells for 10 min. Excess stain was removed with 70% ethanol three times, and lipid droplets were observed under a microscope (Olympus DP71). The absorbance at 600 nm was measured by a plate reader (Molecular Devices, SpectraMax iD3).

MC3T3 cells were stimulated by piezoelectricity and fixed with 4% paraformaldehyde, permeabilized with Triton X-100 (Beyotime, P0096), blocked with goat serum, and incubated with anti-osteopontin primary antibody (Servicebio, GB11500) overnight, followed by FITC-conjugated secondary antibody (Proteintech, SA00003-2). The cytoskeleton was labeled with iFluor™ 555 phalloidin (Yeasen, 40737ES75), and the nucleus was labeled with DAPI (Yeasen, 40728ES03). Cells were observed, and images were taken by a confocal laser microscope (Olympus FV3000).

Osteogenic proteins in MC3T3 cells were extracted with RIPA lysis buffer (Beyotime, P0013B). Total protein was denatured by loading buffer (Beyotime, P0015) and boiling for 5 min. Protein samples were separated on an SDS-PAGE gel and transferred to a PVDF membrane (Millipore). PVDF membranes were blocked with milk and incubated with anti-collagen I antibody (Proteintech, 14695-1-AP), anti-osteopontin antibody (Servicebio, GB11500), anti-RUNX2 antibody (Proteintech, 20700-1-AP), and anti-β-actin antibody (Proteintech, 20536-1-AP) overnight at 4°C. The cPLA2 expression in BMSCs was analyzed using an anti-cPLA2 rabbit polyclonal antibody (Servicebio). PVDF membranes were washed with PBSt and incubated with an HRP-conjugated secondary antibody (Proteintech, SA00001-2). Blots were visualized with ECL solution (Beyotime, P0018S), and images were taken by an imaging system (Clinx ChemiScope S6).

### 4.9 Transcriptomics

TRIzol reagent (Invitrogen, Carlsbad, CA, USA) was used to isolate and purify total RNA, which was measured using NanoDrop ND-1000. Its integrity was evaluated using the Bioanalyzer 2100 (Agilent, CA, USA) and electrophoresis. Two rounds of purification were used to break and separate poly(A) RNA from 1 μg of total RNA using the Magnesium RNA Fragmentation Module (NEB, cat. e6150, USA) and Dynabeads Oligo (dT) 25-61005 (Thermo Fisher, CA, USA). After that, cDNA was reverse-transcribed and U-labeled into second-stranded DNA using *E. coli* DNA polymerase I (NEB, cat. m0209, USA), RNase H (NEB, cat. m0297, USA), and dUTP Solution (Thermo Fisher, cat. R0133, USA). The strand's blunt ends were treated with an A-base and ligated to the indexed adapters with a T-base overhang. The fragments were ligated to single- or dual-index adapters and selected based on size with AMPureXP beads. The ligated products were treated with heat-labile UDG enzyme (NEB, cat. m0280, USA) and amplified using PCR. The resultant cDNA library had an average insert size of 300 ± 50 bp. Finally, using an Illumina NovaSeq™ 6000 (LC-Bio Technology Co., Ltd., Hangzhou, China), we carried out the 2×150 bp paired-end sequencing (PE150) according to the vendor's recommended protocol.

Low-quality bases, unidentified bases with default parameters, and adaptor contamination were eliminated from the reads using the fastp software (https://github.com/OpenGene/fastp). Fastp was then used to confirm the sequence quality. HISAT2 (https://ccb.jhu.edu/software/hisat2) was utilized to map reads to the *Mus musculus* reference genome. Using the default settings, each sample's mapped reads were assembled using StringTie (https://ccb.jhu.edu/software/stringtie). Then, using gffcompare (https://github.com/gpertea/gffcompare), all transcriptomes from all samples were combined to create a comprehensive transcriptome. Following the creation of the final transcriptome, all transcripts' expression levels were estimated using StringTie. FPKM was calculated using StringTie to determine the expression level for mRNAs (FPKM = [total_exon_fragments/mapped_reads (millions) × exon_length (kB)]). The differentially expressed mRNAs were chosen using parametric analysis, fold change > 2, and p-value < 0.05, *n* = 3.

### 4.10 Metabolomics

The collected samples were thawed on ice, and the metabolites were extracted with 80% methanol buffer. The LC-MS system acquired all samples following the machine orders. Firstly, samples were reverse-phase separated by an ACQUITY UPLC system (Waters, Milford, MA, USA) with an ACQUITY UPLC T3 column at a flow rate of 0.3 ml/min, 40°C (100 mm × 2.1 mm, 1.8 μm, Waters, Milford, USA). The mobile phase consisted of solvent A (water, 5 mM ammonium acetate, and 5 mM acetic acid) and solvent B (acetonitrile). Gradient elution conditions were set as follows: 0∼0.8 min, 2% B; 0.8∼2.8 min, 2% to 70% B; 2.8∼5.6 min, 70% to 90% B; 5.6∼6.4 min, 90% to 100% B; 6.4∼8.0 min, 100% B; 8.0∼8.1 min, 100% to 2% B; 8.1∼10 min, 2% B.

A high-resolution tandem mass spectrometer, TripleTOF 6600 (SCIEX, Framingham, MA, USA), was used to detect metabolites in both positive and negative ion modes. The curtain gas was set to 30 PSI, ion source gas 1 was set to 60 PSI, ion source gas 2 was set to 60 PSI, and the interface heater temperature was 500°C. The ion spray voltage floating was set at 5000 V and −4500 V for positive and negative ion modes, respectively. The mass spectrometry data were acquired in IDA mode from 60 to 1200 Da. The survey scans were acquired in 150 ms, and as many as 12 product ion scans were collected if exceeding a threshold of 100 counts per second (counts/s) and with a 1+ charge state. Dynamic exclusion was set for 4 s. The mass accuracy was calibrated for every 20 samples, and a quality control sample (pool of all samples) was acquired after every 10 samples.

The acquired MS data were processed using XCMS software for peak picking, peak grouping, retention time correction, second peak grouping, and annotation of isotopes and adducts. They were converted into mzXML format and then processed by the XCMS, CAMERA, and metaX toolboxes implemented in R software. Intensities of each peak were recorded, and sample names (observations) and ion intensity information (variables) were generated by a three-dimensional matrix containing arbitrarily assigned peak indices (retention time-m/z pairs).

The online KEGG HMDB database was used to annotate the metabolites by matching the exact molecular mass data (*m*/*z*) with a < 10 ppm difference to the database and validated by the isotopic distribution measurements. The intensity of peak data was preprocessed by metaX, where detected features in less than 50% of QC samples or 80% of biological samples were removed, and the peaks with missing values were imputed with the k-nearest neighbor algorithm. PCA was performed for outlier detection and batch effects evaluation. Quality control-based robust LOESS signal correction was fitted to the QC data to minimize signal intensity drift. The metabolic features with > 30% standard deviation were removed across all QC samples. Student t-tests were conducted for statistical analysis, and the p-value was adjusted for multiple tests using an FDR (Benjamini–Hochberg). A fold change > 2 and a p-value < 0.05 were considered significant, *n* = 3. Supervised PLS-DA was conducted through metaX to discriminate variables across groups. A calculated VIP cut-off value of 1.0 was used to select important features.

### 4.11 Rat OVX model and validation

The animal experiment was approved by Fudan University's Animal Care and Use Committee (accession number 2023-156). After anesthesia with ketamine and isoflurane, the bilateral ovaries of 6-week-old female rats were resected and kept for 3 months. The bone mineral density and body composition were assessed by the iNSiGHT DXA system (OsteoSys).

### 4.12 Bone growth stimulated by piezoelectric hydrogel and measured by micro-CT

The kicking force was measured by modifying the method created by Zheng et al. [60]. Briefly, the forelimbs of SD rats were placed on a grid, and the left hindlimb was placed on another grid. A digital force gauge (DAREX) was installed with a flat-top module, and the force generated by the kicking motion was measured and recorded. Each hindlimb was measured three times, and three rats were measured.

After confirming osteopenia due to estrogen deficiency by DXA, 6 OVX rats were randomly assigned to each group by generating random numbers with the RAND function in Excel. After anesthesia with ketamine and isoflurane, the distal femur of OVX rats was exposed, and 3-mm-wide, 3.5-mm-deep defects were created using a trephine. The defects were implanted with 24.5 μl of either RSF hydrogel, MXene/RSF, PVDF/RSF, or MXene/PVDF/RSF, or left vacant without any implantation. The wounds were closed, and rats were given amoxicillin (13.75 mg/kg) and carprofen (5 mg/kg) for 10 days after surgery. The rats were allowed to move freely and fetch food and water when needed. At 6 and 12 weeks, rats were sacrificed by CO_2_, and the femurs were collected and fixed with 5% paraformaldehyde. The defects were scanned by micro-CT (Bruker 1176) with source voltage (kV) = 70 and source current (μA) = 325 using a 1 mm Al filter, and the image pixel size is 17.41 μm. The images were reconstructed by NRecon 1.7.3.0, viewed by CTVox 3.3.0, and sagittal, transverse, and coronal planes were selected. The reconstructed images were analyzed by CTAn 1.17.7.2, and ROIs were defined by manually tracing along the inner surface of the distal femur cortical bone from 150 layers above to 150 layers below the center of the defects. The bone volume, trabecular parameters, and BMD were analyzed by the morphology plug-ins.

### 4.13 Histological exams of bone growth

The femurs were decalcified in EDTA solution until soft enough for sectioning. Then, they were dehydrated, embedded in paraffin, and sectioned to 5 μm thickness. The sections were rehydrated and stained with either H&E (Servicebio, G1005), Masson's Trichrome (Servicebio, G1006), or Safranin O/Fast Green staining solution (Servicebio, G1053). The images were scanned by a slide scanner (PANNORAMIC Flash DESK DX). The lipid droplet parameters were calculated by ImageJ. After thresholding, the number, area, perimeter, and circularity were measured. The BMP2 to PPARγ ratio was calculated by color thresholding, converted to grayscale, and then the intensities were measured. The cPLA2 expression in bone defects was assessed by probing with anti-cPLA2 rabbit pAb (Servicebio, GB11551-50) overnight, rinsing three times with PBS, incubating with goat anti-rabbit IgG(Cy3) (Servicebio, GB27303) for 2 h, and rinsing three times again. The slides were scanned by a slide scanner (PANNORAMIC Flash DESK DX).

## CRediT authorship contribution statement

**Weisin Chen:** Writing – original draft, Investigation, Formal analysis, Data curation, Conceptualization. **Shuyan Zhang:** Investigation, Formal analysis. **Lei Huang:** Data curation. **Yukai Huang:** Investigation. **Minhao Liang:** Formal analysis. **Xin Liu:** Data curation. **Ning Wang:** Software. **Juan Li:** Validation. **Haifeng Liang:** Software. **Dihan Su:** Conceptualization. **Zenggan Chen:** Project administration. **Dong Zhou:** Investigation. **Yulin Li:** Supervision. **Jian Zhang:** Supervision. **Yan Su:** Formal analysis, Data curation. **Yutong Gu:** Validation. **Libo Jiang:** Supervision, Funding acquisition.

## Funding

Thanks to the 10.13039/501100001809National Natural Science Foundation of China (82272457, 82472396, 82673074), the 10.13039/501100003392Fujian Provincial Natural Science Foundation of China (2024D031), the Medical Engineering Fund of 10.13039/501100003347Fudan University (yg2023-27), and the Shanghai Oriental Talent Program.

## Declaration of competing interest

The authors declare that they have no known competing financial interests or personal relationships that could have appeared to influence the work reported in this paper.

## Data Availability

Data will be made available on request.
