# Supplementary material for: Sandwich-structured PVDF/protein piezoelectric hydrogel regulates osteogenesis and adipogenesis through glycerophospholipid metabolism in osteoporotic inflammatory bone defects
Source: Mater Today Bio. 2026 Oct 21;41:103770. doi: 10.1016/j.mtbio.2026.103770 (PMC13715406; doi:10.1016/j.mtbio.2026.103770)
Supplement: Supplementary file 1 [file mmc1.docx]

**Sandwich-Structured PVDF/Protein Piezoelectric Hydrogel Regulates Osteogenesis and Adipogenesis through Glycerophospholipid Metabolism in Osteoporotic Inflammatory Bone Defects—Supplementary Material**

**Figure S1.** The electrical current of RSF, MXene/RSF, and MXene/PVDF(1)/RSF in response to compression. The electrical current was hardly detectable when the piezoelectric material was lacking. The electrical current significantly improved to about 2500 nA when a layer of PVDF membrane was inserted, indicating a potential difference on MXene/PVDF/RSF successfully induced electrical stimulation.

**Figure S2.** Biocompatibility of the extract of piezoelectric hydrogels with different layers of PVDF membranes. (A) The MC3T3 cells were cultured in the extracts from RSF, MXene/RSF, and piezoelectric hydrogels with one, two, and three layers of PVDF membranes. (B) The live-to-dead cell ratio of each group on days 1, 3, and 5. *n* = 3, scale bar represents 500 μm.

**Figure S3**. Cell viability assay, osteogenesis, and calcium deposition are analyzed by the CCK-8 assay, ALP activity, and calcium leaching assay. The cell viability was not significantly affected by TNF-α after 5 days of culture. The ALP activity and calcium deposition were slight but not significantly reduced by TNF-α. Cell viability was analyzed by two-way ANOVA followed by Šídák's multiple comparisons test, *n* = 3. ALP activity and ARS activity were analyzed by student’s t test, *n* = 5. ns = not significant.

**Figure S4.** Immunostaining of BMP2, PPARγ, and the nucleus of the control and OVX femurs. BMP2 was stained red, PPARγ green, and the nucleus blue. The scale bar represents 200 μm. These immunostaining images correspond to Figure 6C.

Element analysis of Ti₃C₂Tx by EDS:

| **Element** | **Wt%** | **At %** |
| --- | --- | --- |
| C | 9.98 | 21.77 |
| O | 13.87 | 22.70 |
| F | 16.01 | 22.07 |
| Al | 1.38 | 1.34 |
| Ti | 58.75 | 32.11 |

**Table S1.** Element analysis of MXene by EDS, provided by the retailer. The ratio of Ti to C is close to 3:2.

**Figure S5.** Micro-CT scan and morphological analysis: (A) Representative images of the No Implant, RSF, MXene/RSF, PVDF/RSF, and MXene/PVDF/RSF scaffolds. (B) BV/TV, BMD, Tb.Th, and Tb.Sp results of each group. *n* = 6, analyzed by ANOVA followed by Tukey’s test, *: p < 0.05, **: p < 0.01, ***: p < 0.001. The red dashed circle indicates the defect area.
